# Contraceptive use and condomless sex among sexually experienced disabled and non-disabled White, Black, and Hispanic female adolescents and young adults aged 18–25 in the United States

**DOI:** 10.21203/rs.3.rs-10318778/v1

**Published:** 2026-08-11

**Authors:** Ruby Siegel, Ilhom Akobirshoev, Justine Wu, Willi Horner-Johnson, Monika Mitra

**Affiliations:** 1 Brandeis University, Lurie Institute for Disability Policy, Waltham, MA, USA; 2 University of Michigan Medical School, Ann Arbor, MI, USA; 3 Oregon Health & Science University, Portland, OR, USA

**Keywords:** race, people with disabilities, contraceptive use, contraception, sexual health

## Abstract

**Background:**

Disabled and racial and ethnic minority women experience barriers to contraceptive access, autonomy, and method preference, yet little research examines these experiences at the intersection of marginalized identities. Our study objective was to examine associations between disability status, race, ethnicity, and contraceptive use among female adolescents and young adults.

**Methods:**

We analyzed data from three consecutive waves of the National Survey of Family Growth (2011–2013, 2013–2015, 2015–2017) focusing on disabled and non-disabled White, Black, and Hispanic females aged 18–25 in the United States. We examined contraceptive use, condomless sex, and method type by disability status, race, and ethnicity, alone and in combination, using logistic regression with robust standard errors to calculate odds ratios with 95% confidence intervals (CI) for binary outcomes and multinomial logistic regression to estimate odds ratios (OR) for multi-category outcomes.

**Results:**

Among females who ever had sex, (N=3,410), at first sex, White non-disabled females were most likely to use short-acting hormonal/long-acting reversible contraceptives (LARC). Black disabled and Hispanic disabled females were significantly more likely to use no method compared to shorting-acting hormonal/LARC. Black and Hispanic non-disabled females had significantly higher rates of condomless sex compared to non-disabled White females. The pattern of racial and ethnic disparities persisted at last sex in the past 12 months and 3 months.

**Conclusions:**

Disparities in contraceptive use were greatest among Black and Hispanic females, both with and without disabilities. The findings suggest the that the combined effects of racism and ableism create unique barriers to equitable sexual and reproductive health services. Healthcare systems, clinicians, and schools must provide disability-competent and culturally responsive interventions for people with disabilities, racial and ethnic minorities, and multiply marginalized individuals.

## BACKGROUND

Adolescent and young adult women are at a formative period in which they may experience sexual desires, have intercourse, and establish relationships with health care providers to learn more about their contraceptive options. Contraception use is central to reproductive autonomy—the ability to make reproductive decisions associated with reproductive health, pregnancy, and childbearing.^1^ Yet women with disabilities and racial and ethnic minority women make reproductive decisions in the context of historical and ongoing bodily coercion that has sought to control contraceptive decision-making.^2–4^

These systems of control may influence contraceptive use and method preference in ways that differ by disability status, race, and ethnicity. For example, a study on racial and ethnic differences on preferred contraceptive features found non-Hispanic (NH) Black and Latina women were more likely than White women to report it “extremely important” to have control over when and whether to use a contraceptive method and to have the ability to become pregnant after discontinuation.^5^ Racial and ethnic minority women have also reported that their concerns have not been taken seriously and that their requests for LARC removal have been denied by providers.^6^ Some disabled women have their contraceptive decisions overseen by a parent or guardian into adulthood.^6–8^ When women with disabilities seek contraception it is sometimes discussed in a way that disabled women cannot understand.^7,9,10^ These barriers to contraception access and autonomy, along with factors such as affordability,^11^ neighborhood deprivation,^12^ insurance status,^12–14^ and awareness;^15,16^ disproportionately effect women with disabilities, racial and ethnic minority women, and racial and ethnic minority women with disabilities. Disabled and racial and ethnic minority women are at the margins of receiving adequate sexual and reproductive health care^17,18^ and information.^19,20^ Some OB-GYN physicians report insufficient training or lacking the confidence to treat patients with disabilities.^21^ Providers also have implicit and explicit racist and ableist bias^21,22^ that can compromise their quality of care. Moreover, most health care facilities are inaccessible for people with disabilities, such as by not having exam tables, signage, and adapted informational materials available for disabled individuals.^23–25^ Proper health care services matter for patient satisfaction. Studies have found that young women from racial and ethnic minority groups who reported higher quality care were more likely to use their preferred method(s) of contraception.^26,27^

The need for accessible contraceptives is compounded by the fact that young women with disabilities experience higher rates of sexual violence compared to non-disabled women,^28–30^ and that Black, Native, and multiracial women are at increased risk of experiencing sexual violence.^31,32^ Women who are sexually abused are more likely to not use contraception in the future^33,34^ and contract STIs.^35^ Thus, access to and use of contraception plays a critical role in STD prevention.

Despite certain improvements in contraception use among women, this practice remains lower than recommended guidelines.^36^ Overall, racial and ethnic minority women are less likely to use contraceptives than White women.^37^ Research is mixed on whether disabled or non-disabled females use contraceptives at a higher rate.^14,16,38^ These disparities likely reflect a combination of structural and attitudinal barriers rooted in ableism, racism, and anti-poverty sentiment as well as preference for contraceptive use and method type.

This study is grounded in intersectionality, which acknowledges that individuals with multiply marginalized identities experience unique forms of discrimination that cannot be understood by examining single identities in isolation.^39^ Intersectionality provides a critical framework for understanding how the combination of disability status, and racial and ethnic minority background may compound vulnerabilities and produce distinct patterns of contraceptive use. While contraceptive use has been studied in disabled and racial and ethnic minority women alone, little is known on contraceptive use of disabled racial and ethnic minority women in combination.

The objective of this study was to examine how disability, race, and ethnicity were associated with contraceptive use and condomless sex among female adolescents and young adults, who have ever had sex, defined as having had sex with a male partner, were sexually active, and experienced voluntary or non-voluntary sex, aged 18–25 in the United States. Specifically, we aimed to answer the following research questions: (1) Does any contraceptive use differ across disabled and non-disabled White, Black, and Hispanic groups at first sex, at last sex in the past 12 months, and at last sex in the past 3 months? (2) Does the type of contraceptive method used differ across these groups? and (3) Does condomless sex differ across these groups?

## METHODS

### Data

We analyzed data from three consecutive cycles of the National Survey of Family Growth (NSFG): (2011–2013, 2013–2015, 2015–2017). The most recent 2017–2019 and 2021–2023 waves were not used because data on forced sex were not included. The NSFG is a nationally representative, cross-sectional survey of the non-institutionalized U.S. household population aged 15–49, conducted by the Centers for Disease Control and Prevention’s National Center for Health Statistics using computer-assisted personal interviewing.^40^ We used the female respondent files, which contained detailed information on demographics, sexual behavior, contraceptive use, and reproductive health. The weighted response rates for female respondents ranged from 66.7–73.4% across the three cycles. Detailed information about the NSFG survey design and methodology is available elsewhere.^40^ Hereafter, when discussing data we will refer to respondents as “females” for consistency with NSFG procedures.

### Study Sample

From the initial sample of 22,995 female respondents aged 15–49, we restricted the analytic sample to females aged 18–25 years (n=4,515) (Figure 1). Our age cut-off reflects the distinct policy environment under the Affordable Care Act (ACA), which since 2010 has allowed young adults to remain on their parents’ health insurance plans until age 26, creating shared insurance access characteristics for this population.^41,42^ We further restricted the sample to female respondents who were sexually active and had ever had sex with a male partner (n=3,729) and those who identified as Non-Hispanic (NH) White, NH Black, or Hispanic of any race (n=3,414). Other racial groups were excluded due to small and heterogenous subgroup sizes that preclude meaningful interpretation. Finally, we excluded respondents who were sterile (n=4), resulting in a final analytical sample of 3,410 females.

### Measures

#### Dependent variables

##### Any contraceptive use.

We assessed any contraceptive method use at three time points using NSFG survey variables: (1) at first vaginal intercourse (derived from SEX1MTHD1–SEX1MTHD4, where codes 1–24 indicated method use and codes 95–96 indicated no method); (2) at last sex in the past 12 months (MTHUSE12; 1 = used a method, 2 = did not use, 95 = never used); and (3) at last sex in the past 3 months (MTHUSE3; 1 = used a method, 2 = did not use, 95 = never used). These outcomes were coded as a binary variable (yes/no). The 12-month and 3-month measures were only asked of respondents who had sex with a male partner in the respective period.

##### Contraceptive method type.

To create categorical method variables, we classified the method reported at each time point using the WHO/CDC tiered-effectiveness hierarchy.^43,44^ When respondents reported multiple simultaneous methods (up to four), we selected the most effective method, consistent with WHO/CDC tiered-effectiveness counseling.^43,44^ Despite the limitations of tiered-effectiveness, pregnancy and STD prevention is an important feature for some contraceptive users. Although we use the tiered-effectiveness hierarchy for data analysis, we examine the findings within patient-centered contraceptive counseling, a model that solicits and honors the values, preferences, and needs of the individual, and does not place judgement on a singular “best” method type.^45^

The contraceptive method type categories included: (1) long-acting reversible contraception (LARC) which includes the intrauterine device (IUD) and contraceptive implant (Nexplanon); (2) short-acting hormonal methods, including the oral contraceptive pill, Depo-Provera (injectable), contraceptive patch, and ring; (3) male condom; and (4) other less effective methods, including withdrawal, fertility awareness–based methods (calendar rhythm, Standard Days, cervical mucus/temperature methods), diaphragm, foam, spermicidal jelly/cream, suppository, sponge, emergency contraception, and other methods. Sterilization was removed due to small cell sizes in the 18–25 age group. Further, at first sex, due to small cells, short-acting hormonal methods and LARC were collapsed into a single category to represent the most effective methods compared to others. Non-users were included as a “no method” category. For multinomial regression, the reference outcome was LARC for the 12-month and 3-month time points, and the combined short-acting hormonal methods/LARC category for first sex.

##### Condomless sex.

We assessed condomless sex as having had sex with a male partner but not reporting condom use among any of the methods listed. Condomless sex was measured as binary variables (yes/no) at first sex, at last sex in the past 12 months, and at last sex in the past 3 months.

#### Independent variables

##### Disability status.

Disability status was measured using the six-item question set from the American Community Survey (ACS), which has been included in the NSFG since 2011.^46^ These included the following six questions: (1) Are you deaf, or do you have serious difficulty hearing?; (2) Are you blind, or do you have serious difficulty seeing, even when wearing glasses?; (3) Because of a physical, mental, or emotional condition, do you have serious difficulty concentrating, remembering, or making decisions?; (4) Do you have serious difficulty walking or climbing stairs?; (5) Do you have difficulty dressing or bathing?; and (6) Because of a physical, mental, or emotional condition, do you have difficulty doing errands alone, such as visiting a doctor’s office or shopping?. We created a binary variable for having a disability, coded 1 if respondents said *yes* to any of these disability questions and 0 if they said *no* to all of them.

##### Race and ethnicity.

We classified respondents as Non-Hispanic (NH) White (hereinafter White), NH Black (hereinafter Black), and Hispanic of any race (hereinafter Hispanic).

##### Cross-classified variable.

To operationalize an intersectional approach, we created a six-category variable combining disability status, race, and ethnicity: 1) White without disability; 2) White with disability; 3) Black without disability; 4) Black with disability; 5) Hispanic without disability; 6) Hispanic with disability.

#### Covariates

The covariates in all our multivariable analyses included age (18–20 years, 21–23 years, 24–25 years); education (less than high school, high school diploma, more than high school); current marital status (married, cohabitating with a partner, not married or cohabitating); health coverage status (yes/no); received checkup for birth control in the past 12 months (yes/no); first vaginal intercourse voluntary (yes/no); household income-to-poverty ratio (<100%, 100–199%, 200–299%, ≥300% of the federal poverty level (FPL); and rural residency (yes/no).

### Statistical analysis

We compared sociodemographic characteristics by disability status within each racial and ethnic group. Differences by disability status were evaluated using chi-squared tests for categorical variables. We also calculated the prevalence and 95% confidence intervals (CI) for each outcome by our cross-classified variable.

For multivariable analyses, binary outcomes (any method use, condomless sex) were modeled using logistic regression with robust standard errors to estimate unadjusted and adjusted odds ratios (OR) and 95% CI,^47^ with White non-disabled females as the reference group. For our categorical outcomes (contraceptive method type), we used multinomial logistic regression to estimate odds ratios (OR) and 95% CI. The reference category was the combined “short-acting hormonal methods/LARC” for first sex and “LARC” methods for most effective contraceptive method used at last sex in the past 12 months, and at last sex in the past 3 months; thus, OR > 1 indicates greater likelihood of using that method category (vs. the reference, i.e. the most effective methods) relative to the reference group.

The adjusted model controlled for age group, educational attainment, marital status, health coverage status, receiving birth control checkup, first vaginal intercourse voluntary, household income-to-poverty ratio, and rural residency. The covariate of receiving checkup for birth control in the past 12 months was only used for outcomes related to sex in the past 3 months and 12 months. First vaginal intercourse voluntary was only used for outcomes related to first sex. All analyses were performed in Stata version 18.0 MP (StataCorp, College Station, Texas). NSFG survey weights for the pooled 2011–2017 dataset (wgt2011_2017) were applied to all analyses to account for the complex multistage probability sampling design and to produce nationally representative estimates.

The Brandeis University Institutional Review Board approved this study.

## RESULTS

### Sample characteristics

Of the 3,410 females aged 18–25, who have ever had sex, approximately 21.9% of the sample reported at least one disability (Table 1). Within the White subgroup, disabled females were less likely to have formal education, less likely to be insured and to receive a checkup for birth control in the past 12 months, less likely to have had voluntary sex at first sex, more likely to live in poverty, and more likely to live in a rural area compared to non-disabled White females. Among Black females, those with disabilities were less likely to have formal education and did not differ based on other sociodemographic characteristics. Among Hispanic females, those with a disability were more likely to be younger, less likely to have formal education, and less likely to have had voluntary sex at first sex and did not differ based on other sociodemographic characteristics.

### Contraceptive use by disability status, race, and ethnicity

Among White females, those with disabilities had significantly lower rates of any contraceptive use at all three time points compared to their non-disabled peers: at first sex (73.3 vs. 85.8%), at last sex in the past 12 months (69.5% vs. 85.7%), and at last sex in the past 3 months (67.7% vs. 85.2%) (Table 2). Black disabled females had slightly lower contraceptive use than non-disabled Black females at first sex (67.2% vs. 70.0%), at last sex in the past 12 months (71.7% vs. 74.0%), and at last sex in the past 3 months (70.8% vs. 74.3%). At first sex, Hispanic disabled females were more likely to use contraception than non-disabled Hispanics (70.4% vs. 67.7%). We did not observe statistically significant differences among Hispanic females at last sex in the past 12 and 3 months. Among all racial and ethnic disabled females, Hispanic disabled females reported the highest rates of contraception use: at last sex in the past 12 months (79.3%) and at last sex in the past 3 months (78.8%).

White females with disabilities had significantly higher rates of condomless sex compared to non-disabled White females at all three points: first sex (38.3% vs. 29.7%), last sex in the past 12 months (69.5% vs. 63.6%); and last sex in the past three months (70.9% vs. 64.6%). Black females with disabilities had significantly higher rates of condomless sex at first sex (44.7% vs. 38.1%) and last sex in the past 12 months (64.9% vs. 61.4%) compared to their non-disabled Black females. Notably, non-disabled Hispanic females had significantly higher rates of condomless sex than disabled Hispanic females at all three points: at first sex (42.8% vs. 38.5%), at last sex in the past 12 months (65.5% vs. 59.4%), and at last sex in past three months (67.8% vs. 63.0%). Among all subgroups, White disabled females reported the highest rates of condomless sex: at last sex in the past 12 months (70.9%) and at last sex in the past 3 months (69.5%). All subgroups were more likely to have condomless sex at their last sex in the past 3 months.

### Prevalence of contraceptive use at the intersection of disability, race, and ethnicity

In our unadjusted analysis, compared to White non-disabled females, White disabled females had significantly lower prevalence (55–64%) of any contraceptive use across all time points (Table 3). Black females—both with and without disabilities—had significantly lower prevalence (50–66%) of any contraceptive use at first sex, and at last sex in the past 12 and 3 months compared to White non-disabled females. Hispanic females—both with and without disabilities—had significantly lower prevalence (61–65%) of contraceptive use only at first sex but not at subsequent time points. Addition of covariates had significant differences for all subgroups at first sex, and White and Black females had significant differences at last sex in the past 12 and 3 months. White disabled females and all other racial and ethnic groups reported higher prevalence of condomless sex at first sex and lowest use at last sex in the past 3 months (Table 3).

#### Contraceptive methods

At first sex, White non-disabled females had the highest prevalence of using the most effective methods, s*hort-acting hormonal method/LARC* (37.3%), versus all other groups (30.8% White disabled; 22.5% Black disabled; 17.1% Black non-disabled; 19.3% Hispanic disabled; 13.5% Hispanic non-disabled) (Table 2). All other racial and ethnic groups and White disabled females had higher prevalence of using *no method* versus the most effective method reference (*short-acting hormonal/LARC)*. Black (50.8%) and Hispanic (50.1%) non-disabled females were more likely to use *condoms* at first sex than White non-disabled females. Similarly, Black (44.3%) and Hispanic (47.1%) disabled females were more likely to use *condoms* at first sex than disabled White females.

At last sex in the past 12 months and last sex in the past 3 months, disabled White (17.6%; 15.2%) and disabled Hispanic (17.0%; 18.3%) females had the highest use of *LARC* compared to all other groups. Black and Hispanic disabled and non-disabled females were more likely to use *condoms* at last sex in the past 12 months and 3 months than non-disabled and disabled White females. The use of *no method* for Black and Hispanic disabled and non-disabled females, and White disabled females persisted at last sex in the past 12 months and 3 months.

Results from our adjusted multinomial logistic regression analysis showed that at first sex, compared to White non-disabled females, White disabled females were significantly more likely to use *no method* (aOR=1.66, 95% CI 1.08–2.55) versus *short-acting hormonal method/LARC* (reference for most effective method). Black non-disabled females were significantly more likely to use *no method* (aOR=3.32, 95% CI 2.17–5.07) and *condoms* (aOR=1.98, 95% CI 1.36–2.88) versus the most effective methods (Table 4). Black disabled females were significantly more likely to use *no method* (aOR=2.45, 95% CI 1.37–4.39) and *other less effective methods* (aOR=0.17, 95% CI 0.03–0.85). Hispanic females without disabilities were significantly more likely to use *no method* (aOR=5.11, 95% CI 3.43–7.64), *other less effective methods* (aOR=3.37, 95% CI 1.54 – 7.38), and *condoms* (aOR=2.83, 95% CI 1.95–4.10). Hispanic disabled females were significantly more likely to use *no method* (aOR=2.52, 95% CI 1.25–5.08) than *shorting-acting hormonal/LARC*.

With LARC as the reference, after adjusting for other covariates, the pattern of racial and ethnic disparities persisted at last sex in the past 12 months and 3 months. White disabled women were significantly more likely to use *shorting-acting hormonal methods* at last sex in the past 12 months (aOR=0.48, 95% CI 0.27–0.84) and *no method* at last sex in the past 3 months (aOR=2.10 CI 95% 1.20–3.68). Black non-disabled females were significantly more likely to use *no method* at last sex in the past 12 months (aOR=2.95 CI 95% 1.75–4.95) and 3 months (aOR=2.83 CI 95% 1.60–5.00). Black disabled females were significantly more likely to use *no method* (aOR=4.19 CI 95% 1.97–8.92) and *condoms* (aOR=2.14 CI 95% 1.08–4.24) in the past 12 months, and *no method* (aOR=4.05 CI 95% 1.80–9.09) in the past 3 months. Hispanic non-disabled females were significantly more likely to use *short-acting hormonal methods* at last sex in the past 12 months (aOR=0.48, 95% CI 0.31–0.75) and past 3 months (aOR=0.52, 95% 0.33–0.83).

In unadjusted analysis, Black disabled (aOR=1.91 95% CI 1.21–3.01), Hispanic non-disabled (aOR=1.77 95% CI 1.39–2.25), White disabled (aOR=1.47 95% CI 1.08–2.00), and Black non-disabled females (aOR=1.46; 95% CI 1.10–1.93) all had significantly higher prevalence of condomless sex compared to White non-disabled females (Table 4). After covariate adjustment, only Black non-disabled females (aOR=1.35 95% CI 1.00–1.82) and Hispanic non-disabled females (aOR=1.57 95% CI 1.20–2.05) had significantly higher rates of condomless sex at first sex. Condomless sex at last sex in the past 12 and 3 months were not significant.

## DISCUSSION

To our knowledge this is the first study to examine contraceptive use across disability status, race, and ethnicity. Specifically, we provided the first nationally representative evidence on contraceptive use among disabled and non-disabled White, Black, and Hispanic female adolescents and young adults in the United States. Among the 3,410 females in our sample, we identified significant disparities in contraceptive use, condomless sex, and method type among females who ever had voluntary and non-voluntary sex.

Disabled and non-disabled Black and Hispanic females were significantly less likely to use *shorting-acting hormonal/LARC* and more likely to use *no method* than White females with and without disabilities. All subgroups were less likely to have condomless sex at first sex and most likely to have condomless sex at last sex in the past 3 months. Black and Hispanic non-disabled females had significantly higher rates of condomless sex at first sex compared to non-disabled White females.

The intersection of disability, race, and ethnicity appears to compound non-use, method type, and less effective method usage for disabled Black and Hispanic females. These findings align with intersectionality, which posits that overlapping systems of oppression including ableism and racism, can exacerbate health disparities.^39^ While LARC is considered the most effective method,^43,44^ we stress the importance of expanding contraceptive access in a way that individuals can choose the method that meets their unique needs and preferences including the ability to choose no method.^45^

These disparities likely reflect structural barriers to education, information, and healthcare, rooted in ableism and racism. Disabled women are less likely to receive formal sex education^29^ and have lower knowledge of contraceptive methods than non-disabled women.^16^ Limited access to disability competent and culturally responsive sexual and reproductive health care,^21,48^ societal assumptions on the sexuality of disabled and racial and ethnic minority women,^3,49^ and challenges such as affordability^11,50^ influence contraception use and access to preferred method(s).

In this study, first sex was the only time we could control for voluntary sex. Contraception use during involuntary sex is different because the opportunity to choose or access contraception may be limited or absent. Rates of contraceptive use and condomless sex may partially be a result of the higher rates of sexual abuse that disabled, and racial and ethnic women have endured.^29,51,52^ Further, prior research has found that early experiences of sexual abuse are associated with engagement in risky sexual behaviors and adult revictimization.^34,53^ These findings highlight the need to distinguish between voluntary and non-voluntary sexual experiences in data collection and when interpreting sexual behavior data among disabled, racial, and ethnic minority individuals

The findings underscore the need to address systemic barriers and identify the underlying mechanisms that restrict access to contraceptives access and choice, sexual health information, and reproductive autonomy for multiply marginalized individuals in the U.S. and across the globe.

### Direction for future research.

Research on factors and sociodemographics that we could not explore due to small sample size, such as Asian or Native populations, or sterilization, also warrants further investigation. This study assessed contraceptive use for sexually active females who had ever had sex with a male partner. These rates may change when focusing on females who have had sex with other females or those without male genitalia. Finally, qualitative research on why individuals decide to use a specific method or no method can offer pathways to meeting the sexual and reproductive health needs and preferences of marginalized populations.

### Policy and practice implications

#### Healthcare policy and access.

The Affordable Care Act (ACA)’s obligation to cover birth control and counseling dramatically improved access to contraception,^41,42,54,55^, with slight reductions in racial/ethnic disparities.^56,57^ Medicaid is the largest public payer of family planning services, covering one in five adult U.S. women of reproductive age (18 to 49 years old).^58^ States that strengthen their contraceptive coverage requirements can make contraception more widely available. For example, some states restrict access to oral contraceptives by failing to cover over-the-counter pills without a prescription or providing patients with a 12-month supply of oral contraceptive pills rather than a 30-day supply.^58,59^ Ongoing threats to Medicaid and the ACA put marginalized populations at greater risk of losing sexual and reproductive health services.^60,61^

#### Sexual health services.

School-based health centers (SBHCs) provide K-12 students access to health services through school-community partnerships. Most patients treated in SBHCs have Medicaid or are uninsured.^62^ Adoption of prohibitive policies at the state, local, or school district level often limits SBHC services. Data from 2010–2011 found that 37% of SBHCs serving middle or high school students dispensed contraceptives, while roughly half of surveyed schools were prohibited from giving out contraceptives.^62^ SBHCs can have positive outcomes, and those that dispense contraceptives are associated with increased contraceptive use among female students.^63^ Greater freedom for SBHCs and participation in efforts such as the Condom Availability Program, which makes condoms freely accessible to all students,^64^ can reduce condomless sex among low-income, disabled, racial, and ethnic minority students.

#### Clinical practice modifications.

Clinicians need the infrastructure to treat their patients with the utmost care including through accessible examination equipment and health information.^65^ Clinician training should teach a patient-centered decision making approach, in which clinicians offer sexual health information while addressing the preferred contraceptive methods and health concerns of their patients.^27^ Training clinicians to provide disability-competent and culturally sensitive services and care is also essential.^66–68^ For adolescents and young adults, these clinical practice guidelines are relevant in public schools, higher education, day centers, institutions, and out-patient care. Individuals should have access to a full range of contraceptive methods—including the ability to choose no method—in a noncoercive manner that meets their values, goals, and reproductive autonomy.^36^

#### Structural and system changes.

Contraceptive use must be viewed in the context of existing social structures and systems rather than seen purely as the responsibility of individual young women. Addressing the intersectional disparities identified in our findings requires systemic changes that address both ableism and racism simultaneously. This should include enforcement of anti-discrimination legislation, such as the Americans with Disabilities Act and Section 1557 of the ACA, policies that address the social determinants of health like insurance coverage and sex education, and expanded abortion and sexual and reproductive health care access in a post-Dobbs landscape;^69^ for example having the right to contraception codified in federal and state law.

### Limitations

Our study has several limitations that should be considered when interpreting the findings. Our disability measure relied on self-reported functional limitations based on the American Community Survey questionnaire. Therefore, those with psychiatric disabilities or chronic health conditions may not be well-represented in the sample^70^ and respondents with cognitive or intellectual and developmental disabilities may have struggled to complete the survey. The NSFG does not collect information on the onset and duration of disability, limiting our ability to determine an individual’s disability status in relation to when the applicable sexual experiences (e.g., first sex, last sex in the past 12 months, etc.) occurred. Given some of the questions’ sensitive nature, respondents might also be prone to recall and social desirability bias. Given that NSFG is a cross-sectional survey, we cannot establish a cause-and-effect relationship between disability status, race, and ethnicity and contraceptive use. Due to sample size limitations, we were unable to examine specific disability types in combination with race and ethnicity. ‘Last sex’ requires that it occur within the past 12 months, but first sex could be at any point. The sample consisted of females aged 18 to 25 at the time of data collection, meaning age at first varied among the study population. This is important because contraceptive use generally increases with age.^37^ Individuals may have different contraceptive usage rates depending on their age of sexual debut.

Black disabled, and particularly Hispanic disabled females, had relatively small sample sizes and this may have affected our ability to reach significant findings within these subgroups. Finally, the NSFG survey groups Hispanic females together despite their heterogenous population and different experiences of systemic racism.^71^

## CONCLUSIONS

Young females with disabilities across all racial and ethnic groups, particularly Black and Hispanic females with and without disabilities, experienced marked disparities in contraceptive use, condomless sex, and method type compared to White non-disabled females. These patterns suggest the that the combined effects of racism and ableism create unique barriers to equitable sexual and reproductive health care.

An intersectional approach is essential to advancing access and autonomy of contraceptives. These findings will require health care systems, clinicians, educators, and schools to enact structural changes to ensure equitable sexual and reproductive health services for disabled, racial and ethnic minorities, and disabled racial and ethnic minority women.

## Figures and Tables

**Figure 1.**
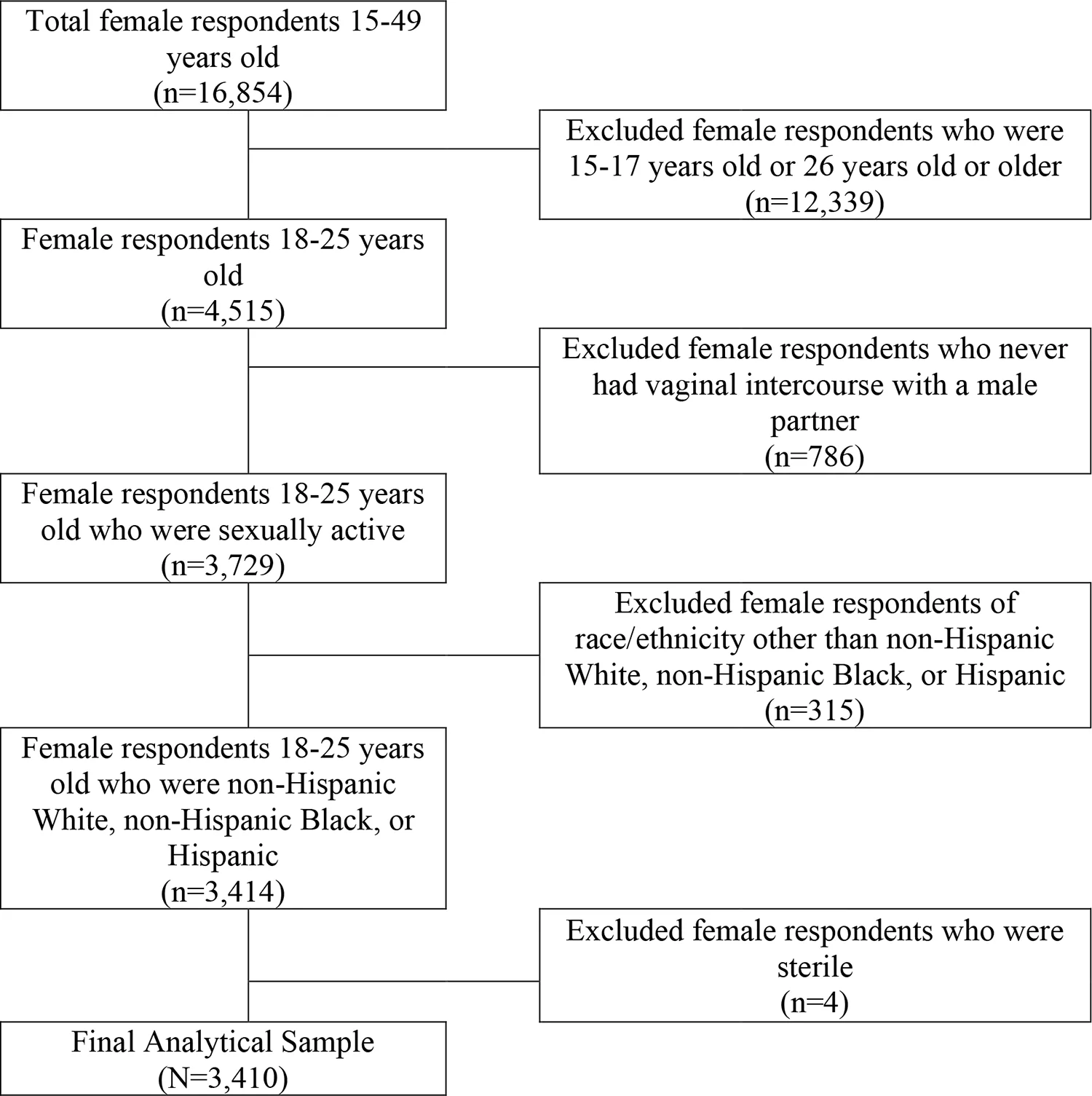
Sample selection flowchart. Data source: National Survey of Family Growth, pooled 2011–2013, 2013–2015, and 2015–2017.

**Table 1.** Sociodemographic characteristics of young women and adolescents by disability status, race, and ethnicity, who ever had sex, (N=3,410)

Characteristics	NH White (n=1,622)			NH Black (n=851)			Hispanic, (n=937)		
	Disability status		p-value	Disability status		p-value	Disability status		p-value
	No (n=1,239)	Yes (n=383)		No (n=677)	Yes (n=174)		No (n=747)	Yes (n=190)	
**Age categories**			0.074			0.501			0.050
18–20	26.5	35.5		30.3	31.1		30.7	42.6	
21–23	42.6	36.4		39.7	45.4		41.0	38.0	
24–25	30.9	28.2		29.9	23.5		28.3	19.3	
**Education**			<0.001			<0.001			0.037
Less than HS	5.2	15.7		10.2	26.9		18.1	28.5	
HS diploma/GED	27.5	41.7		39.5	33.4		32.1	34.1	
More than HS	67.3	42.5		50.3	39.7		49.8	37.4	
**Marital status**			0.157			0.976			0.209
Married	18.5	14.4		6.3	6.7		20.0	13.3	
Cohabitating with a partner	24.8	31.4		16.2	15.4		21.4	19.3	
Not married or cohabitating	56.7	54.2		77.4	77.9		58.6	67.4	
**Currently insured**			0.001			0.807			0.926
No	9.9	19.2		19.7	20.8		28.6	28.1	
Yes	90.1	80.8		80.3	79.2		71.4	71.9	
**Received checkup for birth control last 12 months**			0.002			0.589			0.784
No	50.4	63.0		65.2	62.0		66.8	65.0	
Yes	49.6	37.0		34.8	38.0		33.2	35.0	
**First vaginal intercourse voluntary**			0.006			0.805			0.001
No	3.5	7.5		8.5	9.3		3.6	14.5	
Yes	96.5	92.5		91.5	90.7		96.4	85.5	
**Household income to poverty ratio**			<0.001			0.371			0.089
<100%	21.6	37.1		44.9	54.2		41.0	35.7	
100–199%	20.0	21.0		26.5	18.9		27.5	27.8	
200–299	20.7	16.4		15.1	15.2		11.4	22.7	
300 +	37.7	25.5		13.5	11.7		20.1	13.8	
**Rural residency**			0.001			0.672			0.543
No	80.2	68.7		88.6	89.8		93.2	91.5	
Yes	19.8	31.3		11.4	10.2		6.8	8.5	

Data source: National Survey of Family Growth, pooled 2011–2013, 2013–2015, 2015–2017 cycles. All estimates are weighted to account for the complex survey design. p-values from chi-squared tests comparing disability status within each racial/ethnic group. Note: Ever had sex is defined as ever having had voluntary or non-voluntary vaginal intercourse with a male partner.

**Table 2.** Prevalence of contraceptive use among female adolescents and young adults by disability status, race, and ethnicity, who ever had sex, (N=3,410)

Outcomes	NH White (n=1622)					NH Black (n=851)				Hispanic (n=937)		
	Disability status					Disability status				Disability status		
	No		Yes		No		Yes		No		Yes	
	%	95% CI	%	95% CI	%	95% CI	%	95% CI	%	95% CI	%	95% CI
**Any contraceptive method use**												
At first sex	85.8	83.0–88.2	73.3	66.7–78.9	70.0	64.7–74.8	67.2	57.9–75.3	67.7	62.6–72.4	70.4	59.7–79.2
At last sex, past 12 mos^a^	85.7	82.9–88.1	69.5	61.9–76.2	74.0	68.4–79.0	71.7	61.9–79.8	81.5	76.7–85.5	79.3	70.8–85.8
At last sex, past 3 mos^b^	85.2	82.1–87.8	67.7	59.7–74.7	74.3	67.8–79.9	70.8	59.8–79.8	81.8	76.1–86.3	78.8	69.4–85.8
**Most effective method type at first sex**												
No method	14.2	11.8–17.0	26.7	21.1–33.3	30.0	25.2–35.3	32.8	24.7–42.1	32.3	27.6–37.4	29.6	20.8–40.3
Other less effective	2.6	1.6–4.2	2.8	1.3–5.6	2.2	1.1–4.1	0.4	0.1–1.8	4.1	2.2–7.6	3.9	1.2–12.3
Condom	45.8	41.5–50.1	39.7	33.7–46.1	50.8	45.6–56.0	44.3	34.9–54.0	50.1	44.6–55.6	47.1	37.2–57.2
Short-acting hormonal/LARC^a^	37.3	33.5–41.3	30.8	24.8–37.5	17.1	13.1–22.0	22.5	15.1–32.2	13.5	10.4–17.3	19.3	11.8–29.9
**Most effective method type at last sex, past 12 mos** ^a^												
No method	14.3	11.9–17.1	30.5	23.8–38.1	26.0	21.0–31.6	28.3	20.2–38.1	18.5	14.5–23.3	20.7	14.2–29.2
Other less effective	8.1	6.0–10.8	8.9	5.7–13.5	10.3	7.2–14.6	8.7	4.7–15.5	13.3	9.8–17.7	12.6	6.0–24.7
Condom	16.6	13.7–20.1	18.1	13.6–23.7	26.3	21.2–32.0	29.0	20.3–39.6	27.3	22.5–32.6	27.6	19.1–38.1
Short-acting hormonal	48.7	44.5–52.8	25.0	19.1–31.9	26.9	21.6–33.0	24.8	16.0–36.3	24.0	19.1–29.5	27.0	17.0–40.0
LARC	12.4	10.0–15.3	17.6	11.9–25.2	10.5	7.9–13.8	9.2	5.2–16.0	17.0	12.8–22.1	12.1	7.8–18.3
**Most effective method type at last sex, past 3 mos** ^b^												
No method	14.8	12.2–17.9	32.3	25.3–40.3	25.7	20.1–32.2	29.3	20.3–40.4	18.2	13.7–23.9	21.3	14.2–30.7
Other less effective	8.5	6.2–11.5	9.4	6.1–14.2	10.2	6.7–15.2	8.4	4.3–15.9	13.7	9.8–18.7	14.3	6.7–27.8
Condom	16.1	13.0–19.7	18.7	14.0–24.7	26.1	20.6–32.4	25.5	17.1–36.2	25.4	20.0–31.7	23.5	15.0–34.9
Short-acting hormonal	47.5	43.1–51.9	24.3	18.3–31.6	27.1	21.1–34.2	25.7	16.0–38.7	24.4	19.1–30.6	26.7	16.4–40.3
LARC	13.2	10.5–16.3	15.2	10.2–22.0	10.9	8.0–14.7	11.0	6.2–18.9	18.3	13.7–24.0	14.2	9.2–21.2
**Condom use**												
Condomless at first sex	29.7	25.8–34.1	38.3	32.2–44.9	38.1	33.4–43.2	44.7	34.4–55.5	42.8	37.8–48.0	38.5	29.1–48.7
Condomless at last sex, past 12 mos^a^	63.6	59.9–67.2	69.5	62.8–75.5	60.3	54.2–66.1	59.7	49.1–69.6	65.5	60.3–70.3	59.4	47.5–70.3
Condomless at last sex, past 3 mos^b^	64.6	60.7–68.4	70.9	63.7–77.1	61.4	54.8–67.6	64.9	54.2–74.4	67.8	61.8–73.2	63.0	50.1–74.4

Data source: National Survey of Family Growth, pooled 2011–2013, 2013–2015, 2015–2017 cycles.

Note: Ever had sex is defined as ever having had voluntary or non-voluntary vaginal intercourse with a male partner. In method type at first sex, short-acting hormonal methods and LARC were collapsed into a single “most effective methods” category due to small cell sizes in the disaggregated categories.

When respondents reported multiple methods, the most effective method was selected using the WHO/CDC Tiered-Effectiveness hierarchy.^43,44^

a Excluded 276 respondents who did not have vaginal intercourse with a male partner in the past 12 months.

b Excluded 734 respondents who did not have vaginal intercourse with a male partner in the past 3 months.

**Table 3.** Odds ratios of contraceptive use among female adolescents and young adults, who ever had sex, by disability status, race, and ethnicity, (N=3,410)

Outcomes	NH White		NH Black		NH Black		Hispanic		Hispanic	
	Disability		No Disability		Disability		No Disability		Disability	
	OR	95%CI	OR	95%CI	OR	95%CI	OR	95%CI	OR	95%CI
**Any contraceptive method used at first sex**										
Unadjusted	0.45***	0.32 – 0.65	0.39***	0.29 – 0.52	0.34***	0.22 – 0.53	0.35***	0.26 – 0.47	0.39***	0.24 – 0.65
Adjusted	0.60**	0.41 – 0.87	0.46***	0.33 – 0.65	0.46**	0.29 – 0.73	0.41***	0.29 – 0.57	0.55*	0.33 – 0.94
**Condomless at first sex**										
Unadjusted	1.47*	1.08 – 2.00	1.46**	1.10 – 1.93	1.91**	1.21 – 3.01	1.77***	1.39 – 2.25	1.48	0.92 – 2.38
Adjusted	1.21	0.89 – 1.65	1.35*	1.00 – 1.82	1.59	0.96 – 2.64	1.57**	1.20 – 2.05	1.17	0.69 – 1.96
**Any contraceptive method used at last sex, past 12 mos** ^a^										
Unadjusted	0.38***	0.26 – 0.56	0.47***	0.33 – 0.68	0.42***	0.25 – 0.70	0.73	0.51 – 1.05	0.64	0.38 – 1.08
Adjusted	0.43***	0.28 – 0.65	0.37***	0.25 – 0.54	0.32***	0.18 – 0.57	0.82	0.54 – 1.26	0.62	0.34 – 1.12
**Condomless at last sex, past 12 mos** ^a^										
Unadjusted	1.30	0.92 – 1.85	0.87	0.65 – 1.17	0.85	0.53 – 1.35	1.08	0.83 – 1.42	0.84	0.51 – 1.38
Adjusted	1.28	0.88 – 1.85	1.13	0.84 – 1.53	1.10	0.68 – 1.76	1.21	0.91 – 1.60	1.01	0.61 – 1.65
**Any contraceptive method used at last sex, past 3 mos** ^b^										
Unadjusted	0.36***	0.25 – 0.54	0.50**	0.34 – 0.76	0.42**	0.24 – 0.73	0.78	0.52 – 1.17	0.65	0.36 – 1.15
Adjusted	0.42***	0.27 – 0.64	0.41***	0.27 – 0.63	0.30***	0.16 – 0.58	0.92	0.58 – 1.46	0.69	0.36 – 1.31
**Condomless at last sex, past 3 mos** ^b^										
Unadjusted	1.33	0.92 – 1.93	0.87	0.63 – 1.21	1.01	0.62 – 1.65	1.15	0.85 – 1.56	0.93	0.54 – 1.63
Adjusted	1.30	0.89 – 1.91	1.13	0.82 – 1.56	1.31	0.79 – 2.16	1.26	0.92 – 1.73	1.12	0.66 – 1.93

Data source: National Survey of Family Growth, pooled 2011–2013, 2013–2015, 2015–2017 cycles.

Note: Reference group for all comparisons: NH White females without disabilities. Odds ratios (OR) estimated using logistic regression with robust standard errors. Ever had sex is defined as ever having had voluntary or non-voluntary vaginal intercourse with a male partner.

a Excluded 276 respondents who did not have vaginal intercourse with a male partner in the past 12 months.

b Excluded 734 respondents who did not have vaginal intercourse with a male partner in the past 3 months.

Model for contraceptive use at first sex adjusted for age, education, current marital status, insurance, voluntary sex, household income to poverty ratio, and rural residency status. Model for contraceptive use during sex in the past 12 months or 3 months adjusted for age, education, current marital status, insurance, receipt of checkup for birth control in past 12 months, household income to poverty ratio, and rural residency status.

* p < .05,

** p < .01,

*** p < .001

**Table 4.** Odds ratios of contraceptive method type among female adolescents and young adults, who ever had sex, by disability status, race, and ethnicity, (N=3,410)

Outcomes		NH White		NH Black		NH Black		Hispanic		Hispanic	
		Disability		No Disability		Disability		No Disability		Disability	
		OR	95%CI	OR	95%CI	OR	95%CI	OR	95%CI	OR	95%CI
**Most effective method type at first sex**											
Unadjusted	Short-acting hormonal/LARC	Ref.	Ref.	Ref.	Ref.	Ref.	Ref.	Ref.	Ref.	Ref.	Ref.
	No method	2.28***	1.49 – 3.50	4.61***	3.13 – 6.79	3.83***	2.16 – 6.78	6.29***	4.30 – 9.20	4.04***	1.99 – 8.20
	Other less effective	1.27	0.56 – 2.87	1.78	0.72 – 4.40	0.27	0.06 – 1.31	4.30***	1.89 – 9.78	2.89	0.68 – 12.34
	Condom	1.05	0.74 – 1.50	2.42***	1.67 – 3.52	1.61	0.92 – 2.79	3.02***	2.09 – 4.38	1.99*	1.05 – 3.79
Adjusted	Short-acting hormonal/LARC	Ref.	Ref.	Ref.	Ref.	Ref.	Ref.	Ref.	Ref.	Ref.	Ref.
	No method	1.66*	1.08 – 2.55	3.32***	2.17 – 5.07	2.45**	1.37 – 4.39	5.11***	3.43 – 7.64	2.52**	1.25 – 5.08
	Other less effective	0.96	0.40 – 2.27	1.19	0.44 – 3.27	0.17*	0.03 – 0.85	3.37**	1.54 – 7.38	1.66	0.35 – 7.79
	Condom	1.00	0.71 – 1.42	1.98***	1.36 – 2.88	1.34	0.77 – 2.36	2.83***	1.95 – 4.10	1.69	0.87 – 3.26
**Most effective method type at last sex, past 12 mos** ^a^											
Unadjusted	LARC	Ref.	Ref.	Ref.	Ref.	Ref.	Ref.	Ref.	Ref.	Ref.	Ref.
	No method	1.50	0.80 – 2.82	2.14**	1.33 – 3.44	2.66**	1.29 – 5.47	0.95	0.57 – 1.56	1.48	0.77 – 2.86
	Other less effective	0.77	0.35 – 1.70	1.50	0.84 – 2.68	1.44	0.55 – 3.78	1.20	0.68 – 2.13	1.59	0.57 – 4.48
	Condom	0.77	0.41 – 1.45	1.85*	1.13 – 3.04	2.33*	1.15 – 4.75	1.20	0.73 – 1.95	1.70	0.87 – 3.33
	Short-acting hormonal	0.36***	0.20 – 0.64	0.65	0.41 – 1.02	0.68	0.29 – 1.59	0.36 ***	0.23 – 0.56	0.57	0.30 – 1.08
Adjusted	LARC	Ref.	Ref.	Ref.	Ref.	Ref.	Ref.	Ref.	Ref.	Ref.	Ref.
	No method	1.67	0.92 – 3.04	2.95***	1.75 – 4.95	4.19***	1.97 – 8.92	0.98	0.57 – 1.71	1.67	0.77 – 3.62
	Other less effective	0.88	0.40 – 1.93	1.62	0.87 – 3.02	1.88	0.71 – 4.94	1.29	0.70 – 2.37	1.67	0.57 – 4.84
	Condom	0.83	0.44 – 1.58	1.55	0.93 – 2.58	2.14*	1.08 – 4.24	0.99	0.59 – 1.65	1.33	0.62 – 2.84
	Short-acting hormonal	0.48*	0.27 – 0.84	0.75	0.45 – 1.28	0.90	0.41 – 2.00	0.48**	0.31 – 0.75	0.72	0.40 – 1.32
**Most effective method type at last sex, past 3 mos** ^b^											
Unadjusted	LARC	Ref.	Ref.	Ref.	Ref.	Ref.	Ref.	Ref.	Ref.	Ref.	Ref.
	No method	1.89*	1.03 – 3.47	2.08**	1.23 – 3.54	2.37*	1.12 – 4.99	0.89	0.51 – 1.53	1.33	0.67 – 2.64
	Other less effective	0.96	0.44 – 2.10	1.45	0.76 – 2.79	1.19	0.43 – 3.32	1.16	0.63 – 2.15	1.56	0.54 – 4.50
	Condom	1.01	0.54 – 1.90	1.95*	1.15 – 3.31	1.90	0.92 – 3.94	1.14	0.68 – 1.92	1.36	0.65 – 2.81
	Short-acting hormonal	0.44**	0.25 – 0.79	0.69	0.42 – 1.12	0.65	0.27 – 1.54	0.37***	0.23 – 0.59	0.52*	0.28 – 0.97
Adjusted	LARC	Ref.	Ref.	Ref.	Ref.	Ref.	Ref.	Ref.	Ref.	Ref.	Ref.
	No method	2.10**	1.20 – 3.68	2.83***	1.60 – 5.00	4.05***	1.80 – 9.09	0.91	0.50 – 1.65	1.31	0.59 – 2.88
	Other less effective	1.11	0.52 – 2.37	1.55	0.78 – 3.08	1.72	0.62 – 4.78	1.28	0.66 – 2.47	1.43	0.49 – 4.21
	Condom	1.12	0.62 – 2.04	1.65	0.96 – 2.85	1.94	0.94 – 4.00	0.98	0.57 – 1.70	0.96	0.43 – 2.14
	Short-acting hormonal	0.61	0.35 – 1.06	0.85	0.48 – 1.51	0.83	0.37 – 1.85	0.52**	0.33 – 0.83	0.67	0.36 – 1.25

Data source: National Survey of Family Growth, pooled 2011–2013, 2013–2015, 2015–2017 cycles.

Note: Ever had sex is defined as ever having had voluntary or non-voluntary vaginal intercourse with a male partner.

Reference group for all comparisons: NH White females without disabilities. Categorical method type outcomes estimated using logistic regression and reported as odds ratios with 95% confidence intervals.

For method type at first sex, the reference category is the combined short-acting hormonal/LARC group due to small cell sizes. For method type at last sex in the past 12 and 3 months, the reference category is LARC. OR > 1 indicates greater likelihood of using that method category compared to the reference group.

When respondents reported multiple methods, the most effective method was selected using the WHO/CDC tiered-effectiveness hierarchy.^43,44^

a Excluded 276 respondents who did not have vaginal intercourse with a male partner in the past 12 months.

b Excluded 734 respondents who did not have vaginal intercourse with a male partner in the past 3 months.

Model for contraceptive use at first sex adjusted for age, education, current marital status, insurance, voluntary sex, household income to poverty ratio, and rural residency status. Model for contraceptive use during sex in the past 12 months or 3 months adjusted for age, education, current marital status, insurance, receipt of checkup for birth control in past 12 months, household income to poverty ratio, and rural residency status.

* p < .05,

** p < .01,

*** p < .001

## Data Availability

The date analyzed during the current study are publicly available in the National Center Health Statistics depository: https://www.cdc.gov/nchs/nsfg/nsfg-questionnaires.htm

## Abbreviations

ACA: Affordable Care Act
ACS: American Community Survey
CDC: Centers for Disease Control and Prevention
CI: confidence interval
GED: General Educational Development
HS: high school
IUD: Intrauterine Device
LARC: long-acting reversible contraception
NH: non-Hispanic
NSFG: National Survey of Family Growth
OR: odds ratio
SBHC: School-Based Health Centers
STD: sexually transmitted diseases
STI: sexually transmitted infections
WHO: World Health Organization
